# Actinomycin V Suppresses Human Non-Small-Cell Lung Carcinoma A549 Cells by Inducing G2/M Phase Arrest and Apoptosis via the p53-Dependent Pathway

**DOI:** 10.3390/md17100572

**Published:** 2019-10-09

**Authors:** Shi-qi Lin, Fu-juan Jia, Cai-yun Zhang, Fang-yuan Liu, Jia-hui Ma, Zhuo Han, Wei-dong Xie, Xia Li

**Affiliations:** 1 College of Marine Science, Shandong University, Weihai 264209, China; lsqsd@outlook.com (S.-q.L.); jfj1996@outlook.com (F.-j.J.); caiyun617@outlook.com (C.-y.Z.); fangyuan617@outlook.com (F.-y.L.); sdumjh@hotmail.com (J.-h.M.); hanzhuo1013@gmail.com (Z.H.); wdxie@sdu.edu.cn (W.-d.X.); 2 School of Pharmaceutical Sciences, Shandong University, Jinan 250012, China

**Keywords:** actinomycin, lung cancer, cell cycle arrest, apoptosis, P53

## Abstract

Actinomycin V, extracted and separated from marine-derived actinomycete *Streptomyces sp.*, as the superior potential replacement of actinomycin D (which showed defect for its hepatotoxicity) has revealed an ideal effect in the suppression of migration and invasion in human breast cancer cells as referred to in our previous study. In this study, the involvement of p53 in the cell cycle arrest and pro-apoptotic action of actinomycin V was investigated in human non-small-cell lung carcinoma A549 cells. Results from the 3-(4,5-dimethylthiazol)-2,5-diphenyltetrazolium bromide assay showed that cytotoxic activity of actinomycin V on A549 cells (with wild-type p53) was stronger than the NCI-H1299 cells (p53-deficient). Actinomycin V upregulated both of the protein and mRNA expression levels of p53, p21^Waf1/Cip1^ and Bax in A549 cells. For this situation, actinomycin V decreased the M-phase related proteins (Cdc2, Cdc25A and Cyclin B1) expression, arrested cells in G2/M phase and subsequently triggered apoptosis by mediating the Bcl-2 family proteins’ expression (Bax and Bcl-2). Furthermore, the effects of cell cycle arrest and apoptosis in A549 cells which were induced by actinomycin V could be reversed by the pifithrin-α, a specific inhibitor of p53 transcriptional activity. Collectively, our results suggest that actinomycin V causes up-regulation of p53 by which the growth of A549 cells is suppressed for cell cycle arrest and apoptosis.

## 1. Introduction

p53, a tetramer phosphoprotein, is known as a tumor suppressor and nuclear transcription factor that can mediate several major cellular functions such as gene transcription, DNA synthesis or repair, cell cycle regulation, senescence and cell death [1,2]. Each type of biological function of p53 is largely regulated by transcriptionally regulating various downstream genes. For instance, p53 can induce cell cycle arrest through regulating gene *CDKN1A* which encodes p21^Waf1/Cip1^ expression and can induce apoptotic cell death by increasing the expression of Bax [3]. However, p53 is short-lived and expressed at lower concentrations with a low level of activity in most cell types [4]. Thus, strategies and mechanisms for small molecules that target the p53 pathway, including activating wild-type p53 and temporal combination with mitotic inhibitor, are encouraged and supportive for developing new ways of anti-cancer drug application and therapy [5,6].

Actinomycin D is widely described as a DNA-interacting transcriptional blocker with anti-cancer activity. It is an older chemotherapy drug that has been well used to cure different types of cancers [7,8]. Its use is however limited by its toxicity, especially the hepatotoxicity at high dose. Therefore, attention has been focused on the combination treatment together with other drugs which permit the usage of actinomycin D at a lower concentration [9]. In this connection, reports found that using a low concentration of actinomycin D is very specific for inducing p53 activity and can be utilized for treatment in cooperation with leptomycin B or nutlin-3a to trigger p53 activation and subsequent p53-dependent cellular responses [10].

**Figure 1:**
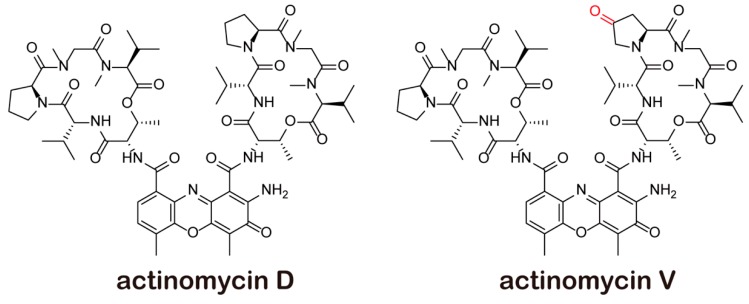
Structure of actinomycins.

Another effective member of the actinomycins is actinomycin V (Figure 1), produced by marine-derived actinomycete *Streptomyces* sp., showing stronger inhibitory effects on various cell lines such as A549 and MCF-7 cells in comparison to actinomycin D, whose cytotoxic effect was not so obvious [11,12,13]. Our previous research also showed that actinomycin V may decrease the snail and slug expressions, suppress the EMT process and reduce the viability of human breast cancer cells [14]. However, the role of actinomycin V in the p53 pathway still remains unclear. In this article, we confirm the G2/M phase arrest and pro-apoptotic effects of actinomycin V in A549 cells, and this action is associated with the p53 activation. These findings may provide a new strategy for the therapy of human p53-positive tumors.

## 2. Results

### 2.1. Cytotoxicity of Actinomycin V on Human Non-Small Lung Carcinoma Cells

To compare the activities of actinomycin V on human non-small-cell lung carcinoma cells, 3-(4,5-dimethylthiazol)-2,5-diphenyltetrazolium bromide (MTT) analyses were carried out to measure the cytotoxicity of actinomycin V on A549 (with wild-type p53), NCI-H1299 (p53-deficient) and normal human bronchial epithelial cells (BEAS-2B). According to Table 1, both actinomycin V and actinomycin D showed greater inhibitory effects on non-small lung carcinoma cells than doxorubicin, which is widely used in clinics for cancer treatment. Surprisingly, actinomycin V showed the remarkable activity on A549 cells while the inhibitory effect in the p53-deficient NCI-H1299 cells was not so ideal. Actinomycin V’s IC_50_ values for 48 h treatment to A549, NCI-H1299 and BEAS-2B were 0.68 ± 0.06 nmol/L, 16.37 ± 1.07 nmol/L and 4.20 ± 0.48 nmol/L, respectively.

### 2.2. Actinomycin V Induces Apoptosis in A549 Cells

**Figure 2:**
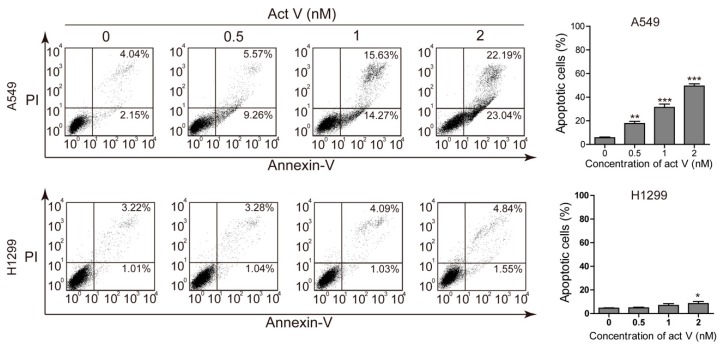
Pro-apoptotic activity of actinomycin V on A549 (with wild-type p53) and NCI-H1299 (p53-deficient) cells. Results from flow cytometry analysis; the quantification of the apoptotic cells after treatment with 0 nmol/L to 2 nmol/L actinomycin V for 24 h. * *p* < 0.05; ** *p* < 0.01; *** *p* < 0.001 vs. the control group.

To study the apoptotic effect of actinomycin V, we chose two non-small lung carcinoma cell lines, A549 and NCI-H1299, to be dual stained with annexin V–fluorescein isothiocyanate (FITC) and propidium iodide (PI) and then measured via flow cytometry (Figure 2). Results showed a strong increase of annexin V-stained cells after actinomycin V treatment in A549 cells. However, the effect of actinomycin V-induced apoptosis was less dramatic in NCI-H1299 cells when compared to the A549 cells. The proportion of annexin V-stained cells increased with the percentages raised from 6.19%, 14.83%, 29.90% to 45.23% (A549) and from 4.23%, 4.32%, 5.12% to 6.39% (NCI-H1299), sequentially.

**Figure 3:**
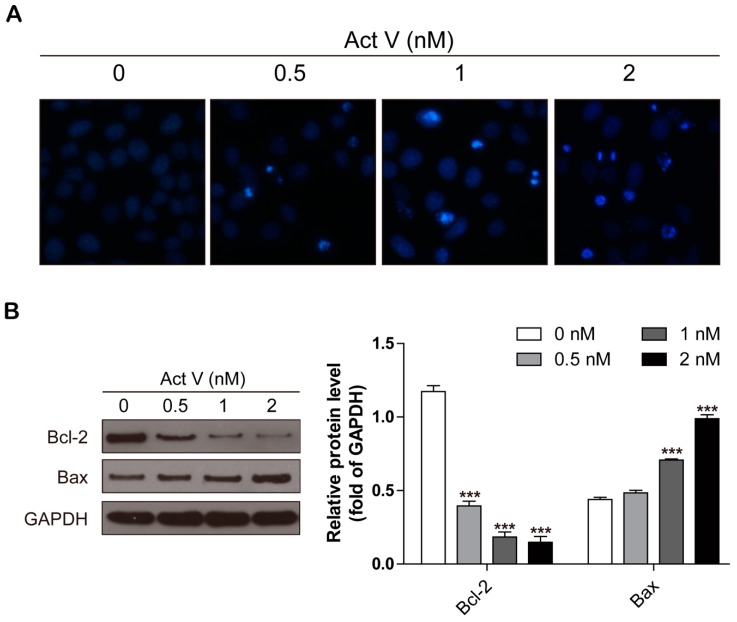
Actinomycin V treatment causing apoptosis in A549 cells. (**A**) Fluorescence micrographs of A549 cells with DAPI staining. Magnification: 100×. (**B**) Western blot showing that actinomycin V induced apoptosis via enhancing Bax and decreasing Bcl-2 protein expressions. *** *p* < 0.001 vs. the control group.

To further confirm the activities of actinomycin V on the morphology of A549 cells during apoptosis, cells were stained with 4’,6-diamidino-2-phenylindole (DAPI) then captured by Cytation 5 Imaging Reader (Bio Tek, Winooski, VT, USA). Compared to the controls in Figure 3A, actinomycin V treatment resulted in obvious apoptotic morphological alterations, involving nuclear condensation and apoptotic bodies formation.

The B-cell lymphoma-2 family proteins, especially the balance between anti-apoptotic protein Bcl-2 and pro-apoptotic protein Bax, exert critical roles in regulating both intrinsic and extrinsic apoptosis. In this present study, we measured the expression levels of Bcl-2 and Bax via Western blot analysis after treatment with actinomycin V for 24 h. Actinomycin V significantly decreased the expression of Bcl-2 and increased that of Bax in a dose-dependent manner (Figure 3B). As a result, we concluded that actinomycin V treatment induced apoptosis in A549 cells.

### 2.3. Actinomycin V Induces G2/M Phase Arrest in A549 Cells

**Figure 4:**
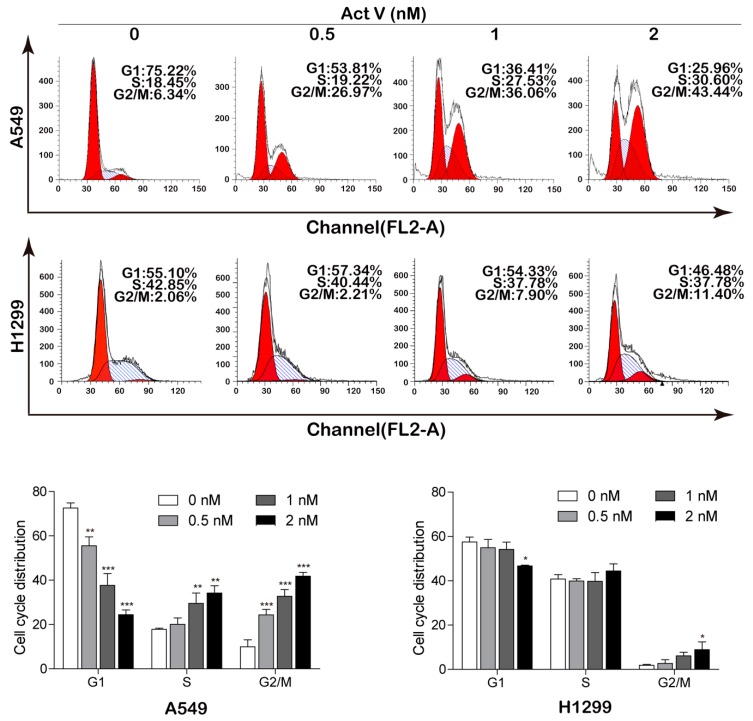
Effects of actinomycin V on cell cycle distribution in A549 (with wild-type p53) and NCI-H1299 (p53-deficient) cells. Flow cytometry analysis detected the cell cycle distribution (each phase presented as G1–S–G2/M: red–stripes–red) of A549 and NCI-H1299 cells after treatment with actinomycin V for 24 h. * *p* < 0.05; ** *p* < 0.01; *** *p* < 0.001 vs. the control group.

Apart from apoptosis, we next examine the cell cycle distribution of A549 cells and NCI-H1299 cells to investigate whether actinomycin V exerted its cytotoxic effects by blocking the cell cycle process. As shown in Figure 4, actinomycin V altered the distribution of the cell cycle in A549 cells while the NCI-H1299 cells were unaffected. After treatment with 0–2 nmol/L actinomycin V for 24 h, the percent of A549 cells arrested in G2/M phase increased along with a decrease of cells in G1 phase. As the control group of A549 cells, only 6.34% of cells were in G2/M phase. However, a remarkable generation in G2/M phase after 0.5 nmol/L to 2 nmol/L actinomycin V treatment (26.97%, 36.06% and 43.44%) was observed in A549 cells.

### 2.4. Actinomycin V Modulates the Expression of M-Phase-Related Proteins

**Figure 5:**
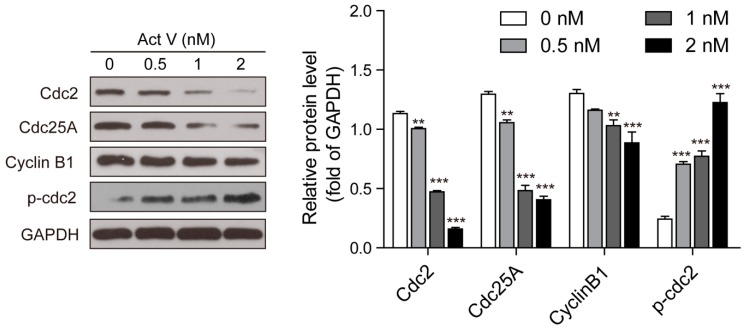
Actinomycin V treatment altered the expressions of M-phase-promoting members. Cells were treated with actinomycin V for indicated hours and the relative protein expressions were detected via Western blotting. ** *p* < 0.01; *** *p* < 0.001 vs. the control group.

The earliest confirmed M-phase-related proteins include Cdc25, Cdc2 and Cyclin B1. Specially, Cyclin B1 may form a complex with Cdc2 to regulate mitosis in eukaryotic cells. Also, the complexes can be activated by Cdc25 to arouse the initiation to mitosis [15]. In this connection, we analyzed the expression levels of these M-phase-related proteins using Western blotting following actinomycin V treatment. In response to actinomycin V treatment, we observed an induction of phosphor-Cdc2 and the reduction of Cyclin B1, Cdc2 and Cdc25A (Figure 5). These results indicated that A549 cells arrested in G2 phase without entering M phase after actinomycin V treatment.

### 2.5. Actinomycin V Induces the Expression of p53 and p21^Waf1/Cip1^

**Figure 6:**
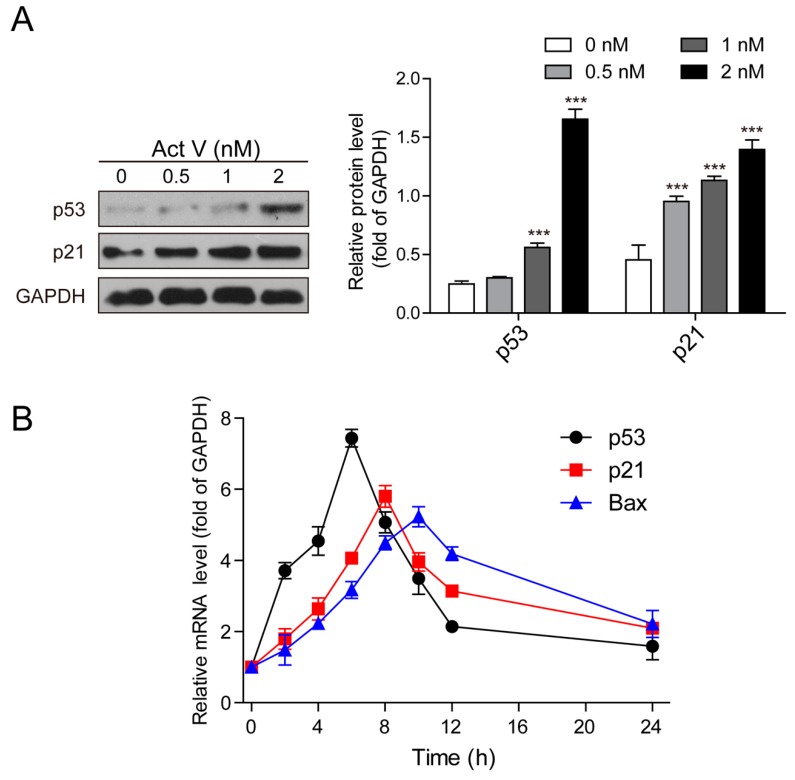
Trends of actinomycin V on p53, p21 ^Waf1/Cip1^ and Bax expression. (**A**) Expressions of relative proteins were measured by Western blotting; (**B**) Relative mRNA expression values were calculated by real-time PCR after 1 nmol/L actinomycin V treatment. The quantity of each mRNA was relative to glyceraldehyde-3-phosphate dehydrogenase (GAPDH) mRNA levels. *** *p* < 0.001 vs. the control group.

To further perceive and recognize the underlying theory of actinomycin V-induced G2/M phase arrest and apoptosis in A549 cells, we tested the expression of p53 and p21^Waf1/Cip1^ using Western blot analysis. Treatment with actinomycin V increased the protein expressions of p53 and p21^Waf1/Cip1^ in A549 cells (Figure 6A).

We also evaluated the effect on the mRNA levels of p53, p21^Waf1/Cip1^ and Bax, another critical downstream mediator of p53. Our results showed that actinomycin V time-dependently increased p53, p21^Waf1/Cip1^, and Bax gene expressions. P53 was activated before p21^Waf1/Cip1^ and Bax, and the activation of p53 mRNA reached the highest levels early, at about 6 h (Figure 6B). Hence, we predict that p53-dependent pathway is associated with G2/M phase arrest and pro-apoptotic effects of actinomycin V.

### 2.6. Inhibition of p53 Blocked Actinomycin V-Induced Cell Cycle Arrest and Apoptosis

**Figure 7:**
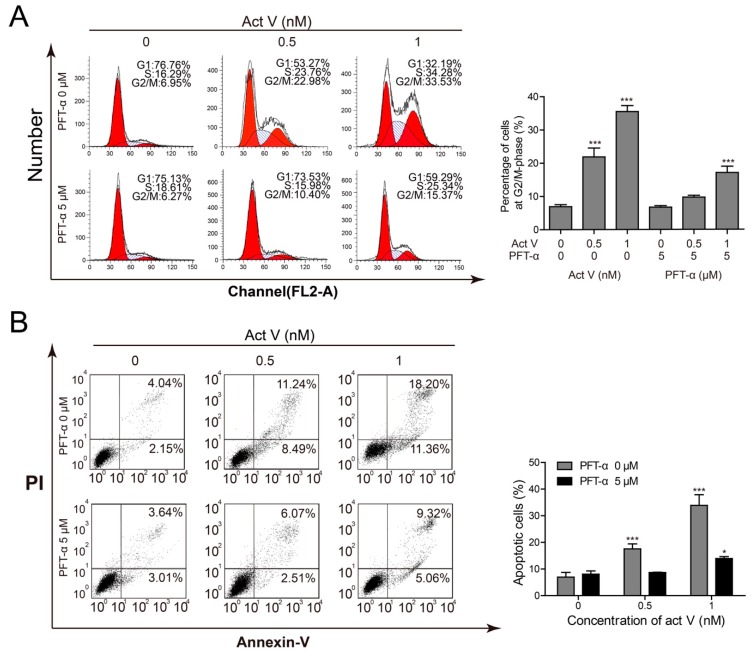
Inhibition of p53 blocked the pro-apoptotic and G2/M phase arrest effect of actinomycin V on A549 cells. Pifithrin-α was used for inhibiting the transcriptional activity of p53; (**A**) cell cycle distribution and (**B**) the quantification of actinomycin V-induced apoptosis were detected via flow cytometry. * *p* < 0.05; *** *p* < 0.01 vs. the control group.

Pifithrin-α (PFT-α) can specifically inhibit the transcriptional activity of p53. Thus, we pre-treated cells with 5 µmol/L PFT-α to find out whether the cell cycle arrest and also the pro-apoptotic effects of actinomycin V were associated with p53 activation. Figure 7A revealed that inhibition of p53 lessened the rate of cell arrest in G2/M phase. Also, the inhibitory effect of PFT-α on p53 led to the reduction of apoptotic cells from 19.73% to 8.58% and 29.56% to 14.38% in the presence of 0.5 nmol/L and 1 nmol/L actinomycin V, respectively (Figure 7B). These data confirmed the involvement of p53 in actinomycin V-mediated G2/M phase arrest and apoptosis.

**Figure 8:**
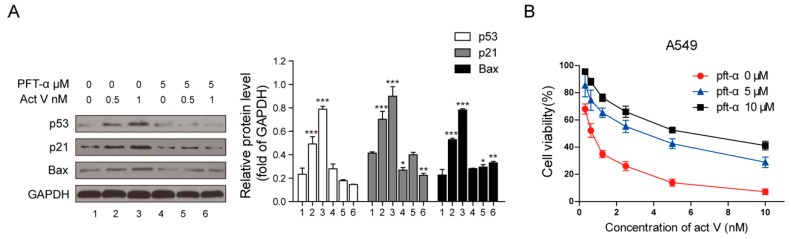
Diminution of p53 changed effects of actinomycin V on the relative protein expressions and the proliferation inhibition toward A549 cells. (**A**) Cells pre-treated with or without 5 µmol/L pifithrin-α for 1 h and exposed to various concentrations of actinomycin V for another 24 h, then analyzed by Western blotting to measure the alteration of relative protein expressions. * *p* < 0.05; ** *p* < 0.01; *** *p* < 0.001 vs. the control group. (**B**) Cells were treated with various concentrations of actinomycin V (with or without 5–10 µM pifithrin-α pretreatment) for 48 h. Data are presented as the mean ± SD (standard deviation) of triplicate experiments.

In addition, after treatment with 5 µmol/L PFT-α, the expressions of p53, p21^Waf1/Cip1^ and Bax decreased significantly when compared to the cells treated with actinomycin V only (Figure 8A). 

### 2.7. Inhibition of p53 Decreased the Cytotoxicity of Actinomycin V

Moreover, we also monitored by MTT assay to determine whether p53 mediated the cytotoxic effect of actinomycin V. Figure 8B illustrates that cells pre-treated with 5–10 µmol/L PFT-α reduced the cytotoxicity of actinomycin V on A549 cells. Hence, we can be certain that the cytotoxicity of actinomycin V on A549 cells is at least partially induced via its p53 protein activation.

## 3. Discussion

Over the past decade, the prospect of new anti-cancer agents research has undergone a tremendous renaissance, especially in the rapidly growing field of small-molecule and biological agents with outstanding clinical activity and lower toxicity when compared to the conventional cytotoxic chemotherapy. Nevertheless, these novel agents are always extremely specific and only valid in a small group of cancers diagnosed as specific genetic lesions or epigenetic alterations. For example, lapatinib, which targets HER2, is efficacious in clinical treatment of breast cancer [16]. The frustration in this field is the inability to discover new agents that focus on the alterations more frequently found in human cancer, key examples include the widespread alteration of the p53 pathway [17]. The p53 protein is a stress-inducible tumor suppressor and nuclear transcription factor. The activation of p53 can trigger several major cellular functions including cell cycle regulation and apoptosis [18]. Moreover, it was confirmed that the p53 protein may malfunction in most human cancers. p53 is inactivated directly (by mutation or deletion) or indirectly (suppress wild type p53 function) in these human cancers [19]. Therefore, the induction of p53 pathway is an effective target for new anti-cancer drug development.

Actinomycin V showed to be more sensitive to the A549 cells when compared to actinomycin D. Our previous study reported that actinomycin V reduced the viability of human breast cancer cells and inhibited the epithelial–mesenchymal transition process. Also, the reduction of snail and slug proteins were associated with the anti-migration and -invasion effects of actinomycin V [14]. In this present study, we chose A549 (wild-type p53) and NCI-H1299 (with deficient p53) cells to study actinomycin V’s functions in p53-mediated cell cycle arrest and apoptosis. 

According to the MTT assay, actinomycin V significantly reduced A549 cells’ viability and the effect was less dramatic in NCI-H1299 cells. Moreover, actinomycin V induced G2/M phase arrest and apoptosis in A549 cells but these induction effects on NCI-H1299 cells were not so obvious. Therefore, we speculate that p53 protein plays an essential role in actinomycin V-induced G2/M phase arrest and apoptosis. In this connection, we confirmed the induction effects of actinomycin V in both the protein and the mRNA expression of p53, and also in p21 ^Waf1/Cip1^ and Bax, two major downstream targets of p53. 

p21 ^Waf1/Cip1^ is described as the cyclin-dependent kinases (CDKs) inhibitor which is critical in not only the G1 to S but also G2 to mitosis transitions [19,20,21]. CDKs usually interact with cyclin proteins to perform their functions in regulating the cell cycle. In particular, the Cdc2/Cyclin B1 complexes are recognized as the M-phase-related protein in eukaryotic cells [22]. Actinomycin V induced phosphor-Cdc2 but reduced Cyclin B1 and Cdc2 expressions, thereby leading to cell cycle arrest in G2 phase without entering the M phase. Moreover, the p53-specific transcriptional inhibitor, PFT-α, partially reversed the effects of actinomycin V on cell cycle arrest and p21 ^Waf1/Cip1^ induction. These results indicated the p53-dependent activation of p21^Waf1/Cip1^ is implicated in the actinomycin V-induced G2/M phase arrest. 

p53 can also affect the transcription of Bax and break the balance between the pro-apoptotic and anti-apoptotic protein, leading to apoptosis [23,24]. Actinomycin V induced Bax and reduced Bcl-2, consequently apoptosis occurred and finally resulted in its proliferation inhibition in A549 cells. Furthermore, the effects of actinomycin V on apoptosis and proliferation inhibition in A549 cells can be partially reversed by the p53-specific transcriptional inhibitor. 

Therefore, actinomycin V inhibits the proliferation of A549 cells through triggering p53 expression and inducing p53-dependent cellular responses, including cell cycle arrest and apoptosis. These findings will be useful for new anti-cancer drug development. However, the limitation is that the selectivity index between cancer and normal cell lines still not ideal. For this reason, toxicity investigation is strongly warranted for actinomycin V to be further developed as marine-derived anti-cancer agent.

## 4. Materials and Methods 

### 4.1. Reagents, Cell Lines and Cell Culture

Actinomycin V (>98%) and actinomycin D (>98%) were dissolved and their concentration adjusted by dimethyl sulfoxide (DMSO) when required while the control group was treated with DMSO only. MTT and DAPI were acquired from Sigma-Aldrich Crop. (St. Louis, MO, USA). Annexin V-FITC Apoptosis Detection Kit was obtained from BD Biosciences (San Jose, CA, USA). Phosphor-Cdc2, Cdc2, Cdc25A, Cyclin B1, p53 and p21^Waf1/Cip1^ were supplied by Cell Signaling Technology (CST, Inc, Beverly, MA, USA). Bcl-2, Bax and GAPDH antibodies were supplied by Abcam, Inc. (Cambridge, MA, USA). Propidium iodide and Pifithrin-α (PFT-α) were purchased from Beyotime Institute of Biotechnology (Shanghai, China). RNeasy mini kit and Rever Tra Ace qPCR RT kit were purchased from QIAGEN (Hilden, Germany) and Toyobo (Osaka Prefecture, Japan), respectively. 

Human non-small-cell lung carcinoma cell lines A549, NCI-H1299 (p53-deficient) and normal human bronchial epithelial cells BEAS-2B were obtained from the Shanghai Institute for Biological Sciences (SIBS), Chinese Academy of Sciences (Shanghai, China) and we cultured the cells according to the supplier’s instructions.

### 4.2. MTT Assay

A549 cells were treated with actinomycin V at the indicated concentration range from 0 to 20 nmol/L in the absence or presence of 5 µmol/L PFT-α pretreatment for 1 h. Then, cytotoxic effects of actinomycin V with or without PFT-α pre-treatment were evaluated by MTT assay as is previously mentioned [14]. In particular, we set the negative group with equal volumes of DMSO to calculate IC_50_ values. 

### 4.3. DAPI Staining

DAPI staining assay, as previously described [25], was used to observe the change of the nucleus after 24 h treatment with actinomycin V among A549 cells.

### 4.4. Apoptosis Detection via Flow Cytometry

The apoptosis rate of A549 and NCI-H1299 cells through 24 h treatment with actinomycin V with or without PFT-α pretreatment was evaluated by Annexin V-FITC and PI double staining according to our previous description [25].

### 4.5. Cell Cycle Distribution

Indicated concentrations of actinomycin V (with or without 5 µmol/L PFT-α pretreatment) were used to treat A549 and NCI-H1299 cells for 24 h. As previously described [25], cells stained with PI were tested by flow cytometry in order to measure the cell cycle distribution, and the data were analyzed using the Modfit program.

### 4.6. Western Blot Analysis

After pre-treating with or without 5 µmol/L PFT-α, cells were incubated with indicated concentrations of actinomycin V for 24 h. Western blot analysis, which we performed as previously described [14], was performed to measure the expressed situation of the indicated protein.

### 4.7. Real-Time PCR Analysis

Real-time PCR assay, as described in our previous work [14], was carried out to measure the mRNA levels of the *p53*, *p21^Waf1/Cip1^* and *Bax* genes. We designed all needed primers using primer premier 5 and synthesis by Sangon Biotech Co Ltd. (Shanghai, China) for the *p53* gene (forward primer: 5’-GTTTCCGTCTGGGCTTCT-3’ and reverse primer: 5’-CCTCAGGCGGCTCATAG-3’), *p21^Waf1/Cip1^* (forward primer: 5’-CCCGTGAGCGATGGAAC-3’ and reverse primer: 5’- AAATCTGTCATGCTGGTCTGC-3’) and *Bax* gene (forward primer: 5’-TCAACTGGGGCCGGGTTGTC-3’ and reverse primer: 5’-CCTGGTCTTGGATCCAGCC-3’). *GAPDH* gene (forward primer: 5’-CATCAAGAAGGTGGTGAAGCAGG-3’ and reverse primer: 5’-TCAAAGGTGGAGGAGTGGGTGTCGC-3’), was used as a control to calculate the relative mRNA levels. 

### 4.8. Statistical Analysis

Data are presented as mean ± standard deviation from triplicate experiments. All data were analyzed using one-way analysis of variance (ANOVA) followed by Tukey’s multiple comparison test (conducted using GraphPad Prism 5.01). * *p* < 0.05; ** *p* < 0.01; *** *p* < 0.001 vs. the control group.

## Figures and Tables

**Table 1:** Cytotoxicity of actinomycins and adriamycin on different cell lines.

Compounds IC50 (nmol/L)	BEAS-2B	A549	NCI-H1299
Actinomycin V	4.20 ± 0.48	0.68 ± 0.06	16.37 ± 1.07
Actinomycin D	16.52 ± 1.03	12.84 ± 0.72	26.64 ± 1.32
Doxorubicin	1091.00 ± 11.01	941.60 ± 15.37	1546.33 ± 41.09

Cytotoxicity of actinomycins and doxorubicin on BEAS-2B, A549 and NCI-H1299 cells. Cells were exposed to varying concentrations of compounds then the IC_50_ values (concentration resulting in 50% inhibition of cell growth) were measured via 3-(4,5-dimethylthiazol)-2,5-diphenyltetrazolium bromide (MTT) assay after 48 h incubation. Equal volumes of dimethyl sulfoxide (DMSO) were used as the negative control. We presented the data as the mean ± SD (standard deviation) of triplicate independent examinations.
